# The Changes of T-Wave Amplitude and QT Interval Between the Supine and Orthostatic Electrocardiogram in Children With Dilated Cardiomyopathy

**DOI:** 10.3389/fped.2021.680923

**Published:** 2021-07-06

**Authors:** Cheng Tan, Xiuying Yi, Ying Chen, Shuangshuang Wang, Qing Ji, Fang Li, Yuwen Wang, Runmei Zou, Cheng Wang

**Affiliations:** ^1^Department of Pediatric Cardiovasology, Children's Medical Center, The Second Xiangya Hospital, Central South University, Changsha, China; ^2^Department of Pediatrics, The Affiliated Zhuzhou Hospital, Xiangya School of Medicine, Central South University, Zhuzhou, China

**Keywords:** electrocardiography, supine position, orthostatic position, children, dilated cardiomyopathy

## Abstract

**Objectives:** Electrocardiogram (ECG) can be affected by autonomic nerves with body position changes. The study aims to explore the ECG changes of children with dilated cardiomyopathy (DCM) when their posture changes.

**Materials and methods:** Sixty-four children diagnosed with DCM were recruited as research group and 55 healthy children as control group. T-wave amplitude and QT interval in ECG were recorded, and their differences between supine and orthostatic ECG were compared in both groups. Subsequently, the children with DCM were followed up and the differences before and after treatment compared.

**Results:** ① Comparisons in differences: Differences of T-wave amplitude in lead II and III, aVF, and V_5_ and differences of QT interval in lead II, aVL, aVF, and V_5_ were lower in the research group than in the control group. ② Logistic regression analysis and diagnostic test evaluation: The differences of T-wave amplitude in lead III and QT interval in lead aVL may have predictive value for DCM diagnosis. When their values were 0.00 mV and 30 ms, respectively, the sensitivity and specificity of the combined index were 37.5 and 83.6%. ③ Follow-up: In the response group, the T-wave amplitude difference in lead aVR increased and the difference of QT interval in lead V_6_ decreased after treatment. In the non-response group, there was no difference before and after treatment. When the combined index of the differences of T-wave amplitude difference in lead aVR and QT interval difference in lead V_6_, respectively, were −0.05 mV and 5 ms, the sensitivity and specificity of estimating the prognosis of DCM were 44.4 and 83.3%.

**Conclusions:** The differences of T-wave amplitude and QT interval may have a certain value to estimate DCM diagnosis and prognosis.

## Introduction

Dilated cardiomyopathy (DCM) is a very serious structural heart disease, the most common in childhood cardiomyopathy, accounting for about 60%. The causes of DCM are diverse, and most of the DCM cases are difficult to determine. Common causes include gene mutations and inflammation. It is characterized by progressive cardiac enlargement, progressive heart failure, and progressive malignant arrhythmia (1). The clinical prognosis of DCM in children is unfavorable, and the mortality rate is high. The mortality rate in the first year after diagnosis reaches 21%, and arrhythmia and heart failure are common causes of death (2–4). When heart failure occurs in DCM children, the sympathetic nervous system is activated. The system can initially maintain relative stability of cardiovascular activity. However, they are also directly toxic substances to cardiomyocytes. Their long-term activation prompts the myocardial cell damage, apoptosis, and myocardial fibrosis, resulting in cardiac decompensation and progression of heart failure (5).

Electrocardiogram (ECG) is an important test method for diagnosing cardiovascular disease, which is non-invasive, simple, and reproducible. The waveform in ECG can reflect ventricular depolarization or/and repolarization. Any factor that affects the ventricular depolarization and repolarization can lead to their changes, such as the excitation of autonomic nerves, myocardial ischemia, myocardial infarction, and electrolyte disorder. Studies have shown that T-wave and QT interval changes are significantly correlated with the risk of fatal arrhythmia and death (6–11). The changes of position are a common way to make the autonomic nerves excited. However, there is no report on the changes of T-wave amplitude and QT interval between the supine and orthostatic ECG in DCM. This article retrospectively analyzes their differences to explore its diagnostic and prognostic value in DCM.

## Methods

### Participants

This was a retrospective study. All the cases came from The Second Xiangya Hospital, from October 2008 to July 2018. The research group had only 64 children with DCM who had the supine and orthostatic ECG. The control group had only 55 healthy children who were matched by gender and age and had supine and orthostatic ECG. The diagnosis of DCM met World Health Organization/International Heart Federation diagnostic standards (12). Statistical differences were not found in male/female (24/31 vs. 29/35, χ^2^ = 0.03, *P* > 0.05) and age [7.00 (5.00–10.00) years vs. 7.50 (5.00–11.00) years, *Z* = −0.224, *P* > 0.05]. The LVEF value, FS value, and inner diameter values of the left atrium and left ventricle of children with DCM were 59.00 (46.50~64.00)%, 31.00 (23.00~35.00)%, 24.00 (21.25~31.50) mm, and 43.50 (39.50~50.00) mm, respectively. Among the research group, 21 children with DCM were followed up for 3-25 months (average 10 ± 6 months); others were lost to follow-up. According to changes in LVEF and clinical symptoms (exercise tolerance, spirit, etc.), some children were divided into the response group (LVEF increased by more than 5% and clinical symptoms improved after treatment), and the others were the non-response group.

### Treatment for the Follow-Up Children

Comprehensive treatment measures such as diuretics, beta blockers, cardiotonic drugs, and improvement of myocardial metabolism are taken for children with DCM (13).

### Operation of ECG

The ECG was traced according to the methods reported in the past (14). The subjects were required to stop any kind of cardiovascular active drugs for at least five half-life periods and other drugs that affect autonomic nervous function. The ECG was recorded with the SR-1000A ECG Automatic Analyzer produced by Zhongshan, Guangdong Province, China. The participants were needed to stand for 5 min after recording the supine ECG, and then the orthostatic ECG was recorded at the same position of the electrode. The sampling did not use filtering equipment. Thirty seconds of stable waveforms were collected and stored in the computer to create case files. ECG was recorded at 25 mm/s paper speed and 10 mm/mV gain setting.

### Measurement of ECG

The initial point of the Q-wave was taken as the reference baseline. Measurement of the QT interval was as follows: the time from the starting point of the QRS complex to the end point of the T-wave, which was an intersection of the T-wave descending branch and measurement baseline. The U-wave was not included in the QT interval, and when the T-wave was a biphasic T-wave, the end time was when the T-wave returned to baseline (15, 16). Measurement of T-wave amplitude was as follows: the positive amplitude was the vertical distance from the peak of the waveform to the horizontal baseline edge, the negative amplitude was the vertical distance from the bottom of the waveform to the horizontal baseline edge, and the bipolar amplitude was the algebraic sum of the positive and negative T-waves. The difference value was the value of supine ECG minus the value of orthostatic ECG. The difference value between before and after treatment was the difference value after treatment minus the difference value before treatment.

### Statistical Analysis

SPSS 23.0 statistical software (IBM Corp, Armonk, New York, USA) was used for all data analyses. Data of quantitative variables were expressed as median (interquartile range). The rank-sum test of Mann–Whitney *U* was performed between two groups, and the Wilcoxon signed rank-sum test was performed before and after treatment. Data of categorical variables were expressed as frequencies. χ^2^ tests were used for comparisons of categorical variables. With α = 0.05 as the test level, *P* < 0.05 was considered statistically significant.

## Results

### Comparisons of T-Wave Amplitude and QT Interval Between the Supine and Orthostatic ECG

**Figure 1:**
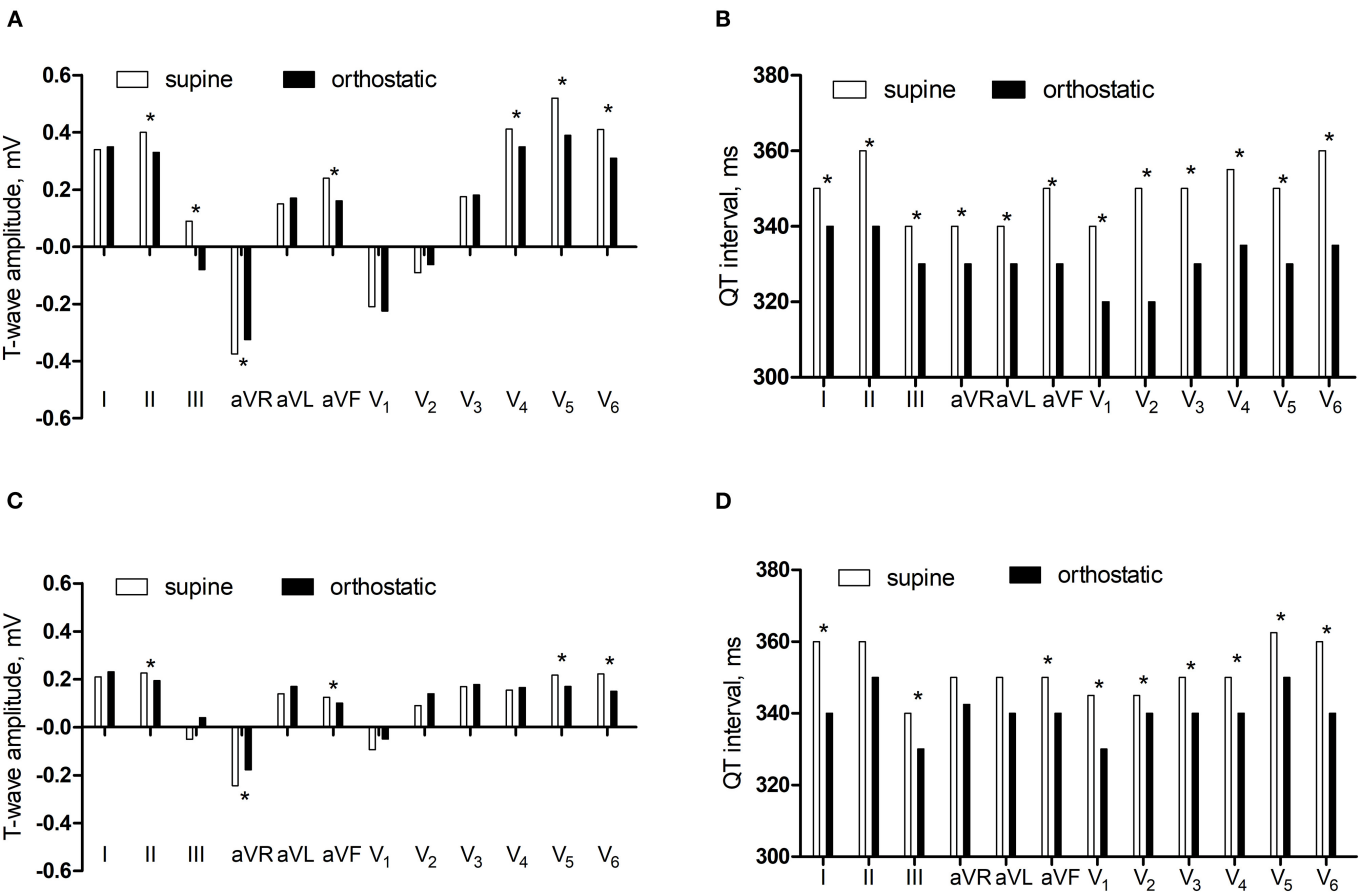
The comparisons of T-wave amplitude and QT interval between supine and orthostatic ECG. **(A)** The T-wave amplitude in the control group. **(B)** The QT interval in the control group. **(C)** The T-wave amplitude in the research group. **(D)** The QT interval in the research group. ^*^P < 0.05.

In the control group, the amplitudes of T-wave in lead II and III, aVR, aVF, V_4_, V_5_, and V_6_ were lower and the QT intervals in lead I and II, III, aVR, aVL, aVF, V_1_, V_2_, V_3_, V_4_, V_5_, and V_6_ were shorter in orthostatic ECG than in supine ECG (*P* < 0.05). In the research group, the amplitudes of T-wave in lead II, aVR, aVF, V_5_, and V_6_ were lower and the QT intervals in lead I and III, aVF, V_1_, V_2_, V_3_, V_4_, V_5_, and V_6_ were shorter in orthostatic ECG than in supine ECG (*P* < 0.05) (Figure 1).

### Comparisons of T-Wave Amplitude and QT Interval Between Control Group and Research Group

**Figure 2:**
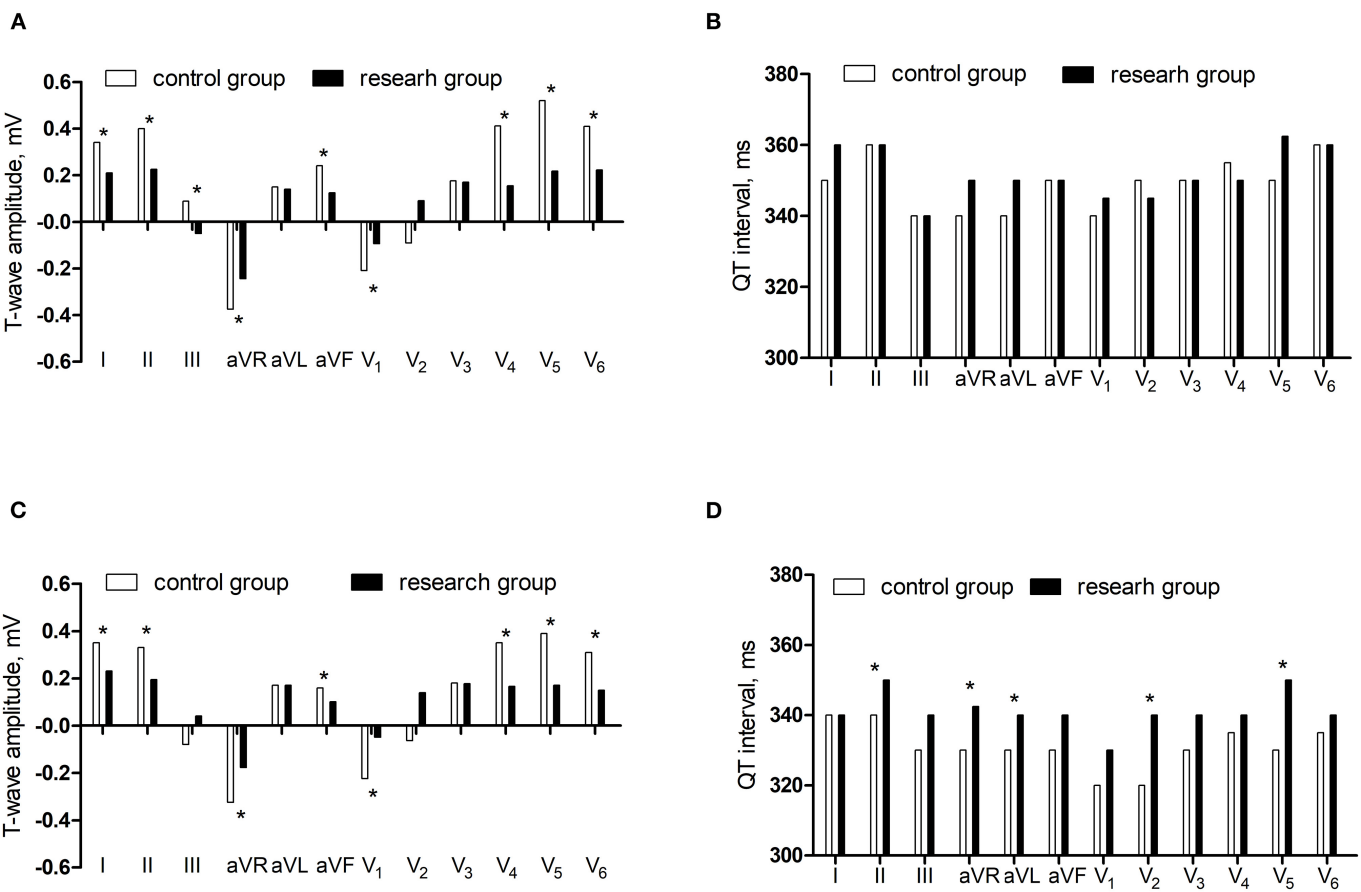
The comparisons of T-wave amplitude and QT interval between control group and research group. **(A)** The T-wave amplitude in the supine ECG. **(B)** The QT interval in the supine ECG. **(C)** The T-wave amplitude in the orthostatic ECG. **(D)** The QT interval in the orthostatic ECG.*P < 0.05.

In supine ECG, the T-wave amplitudes in lead I, II, and III, aVR, aVF, V_1_, V_4_, V_5_, and V_6_ were lower in the research group than in the control group (*P* < 0.05) and there was no significant difference in the QT interval in the two groups (*P* > 0.05). In orthostatic ECG, the T-wave amplitude in lead I and II, aVR, aVF, V_1_, V_4_, V_5_, and V_6_ were lower in the research group than in the control group (*P* < 0.05) and the QT interval in lead II, aVR, aVL, V_2_, and V_5_ were longer in the research group than in the control group (*P* < 0.05) (Figure 2).

### Comparisons of the Differences of T-Wave Amplitude and QT Interval

**Figure 3:**
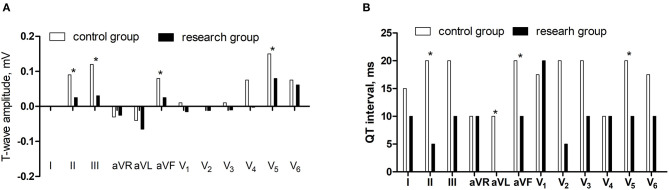
The comparisons of differences of T-wave amplitude and QT interval between supine and orthostatic ECG. **(A)** The differences of T-wave amplitude. **(B)** The differences of QT interval.*P < 0.05.

Compared with the control group, the differences of T-wave amplitude in lead II and III, aVF, and V_5_ decreased and the differences of QT interval in lead II, aVL, aVF, and V_5_ reduced in the research group (*P* < 0.05) (Figure 3).

### Logistic Regression Analysis and Evaluation of the Cutoff Point

To rule out the influence of confounding factors, logistic regression analysis was conducted on the T-wave amplitude differences in leads II, III, aVF, and V_5_ and the QT interval differences in lead II, aVL, aVF, and V_5_. The differences of T-wave amplitude in lead III and QT interval in lead aVL had statistic significance (*P* < 0.05) (Table 1).

**Table 1:** Logistic regression analysis of T-wave amplitude differences in leads II and III, aVF, and V_5_ and QT interval differences in lead II, aVL, aVF, and V_5_.

	**Regression Coefficient**	**Wald**	***P*-value**	**OR**
**T-wave amplitude differences**				
II	3.976	1.707	0.191	53.316
III	−8.307	7.914	0.005	0.000
aVF	3.392	0.672	0.412	29.740
V_5_	−3.049	3.159	0.076	0.047
**QT interval differences**				
II	−0.014	2.908	0.088	0.986
aVL	−0.025	3.884	0.049	0.976
aVF	0.006	0.31	0.578	1.006
V_5_	−0.009	0.776	0.378	0.991

**Figure 4:**
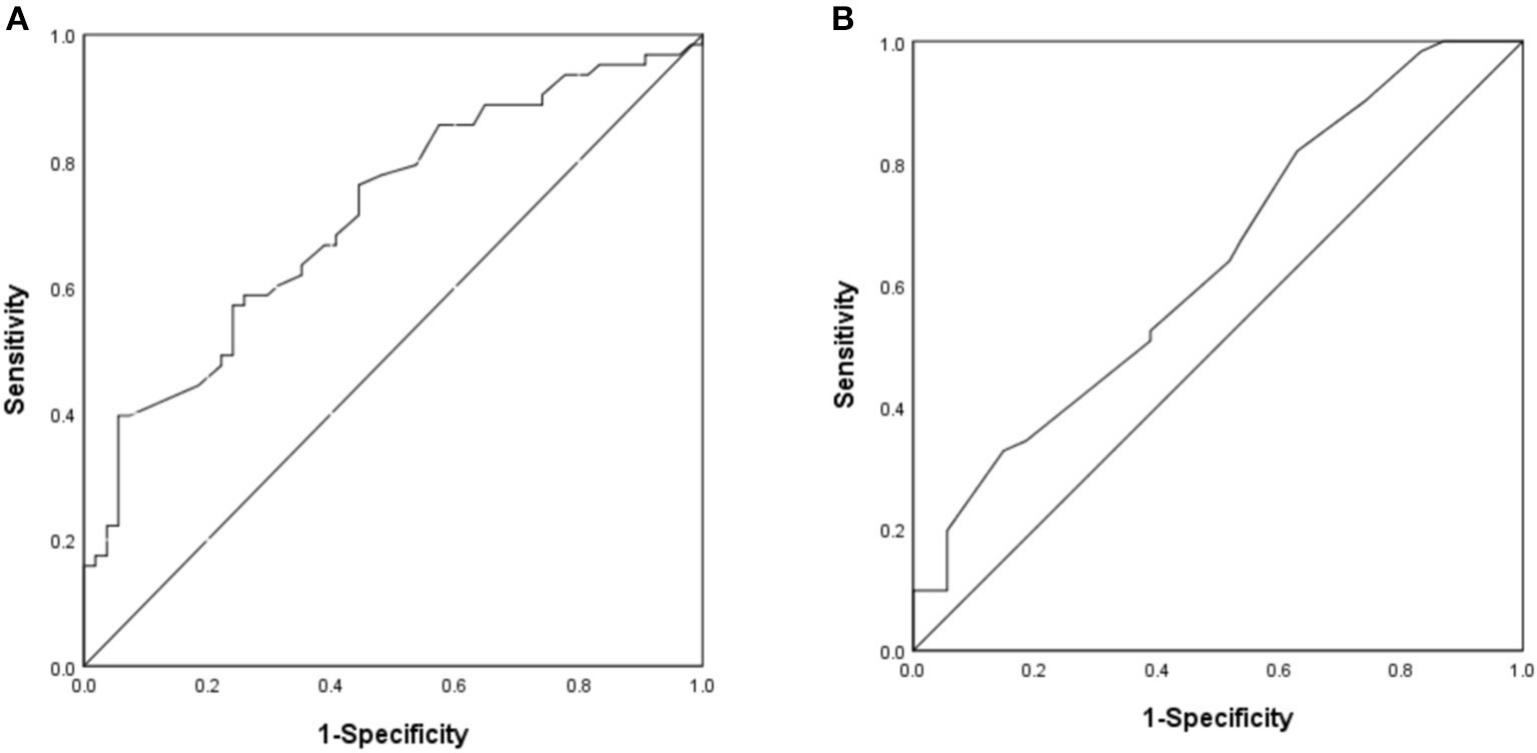
The ROC curve of predictive value on DCM diagnosis. **(A)** The difference of T-wave amplitude in lead III. **(B)** The difference of QT interval in lead aVL.

The area under the ROC curve of the differences of T-wave amplitude in lead III and QT interval in lead aVL were 0.715 and 0.635 (*P* < 0.05), suggesting that may have estimated the value for DCM diagnosis. The best cutoff value of T-wave amplitude difference in lead III was −0.01 mV, the sensitivity and specificity were 39.7 and 94.4%. The best cutoff value of QT interval difference in lead aVL was 25 ms, the sensitivity and specificity were 82.0 and 37.0% (Figure 4).

For clinical application, we set the values of the differences of T-wave amplitude in lead III and QT interval in lead aVL which were 0.00 mV and 30 ms, respectively. The sensitivity and specificity of the combined index were 37.5 and 83.6%, respectively.

### Prognosis

**Figure 5:**
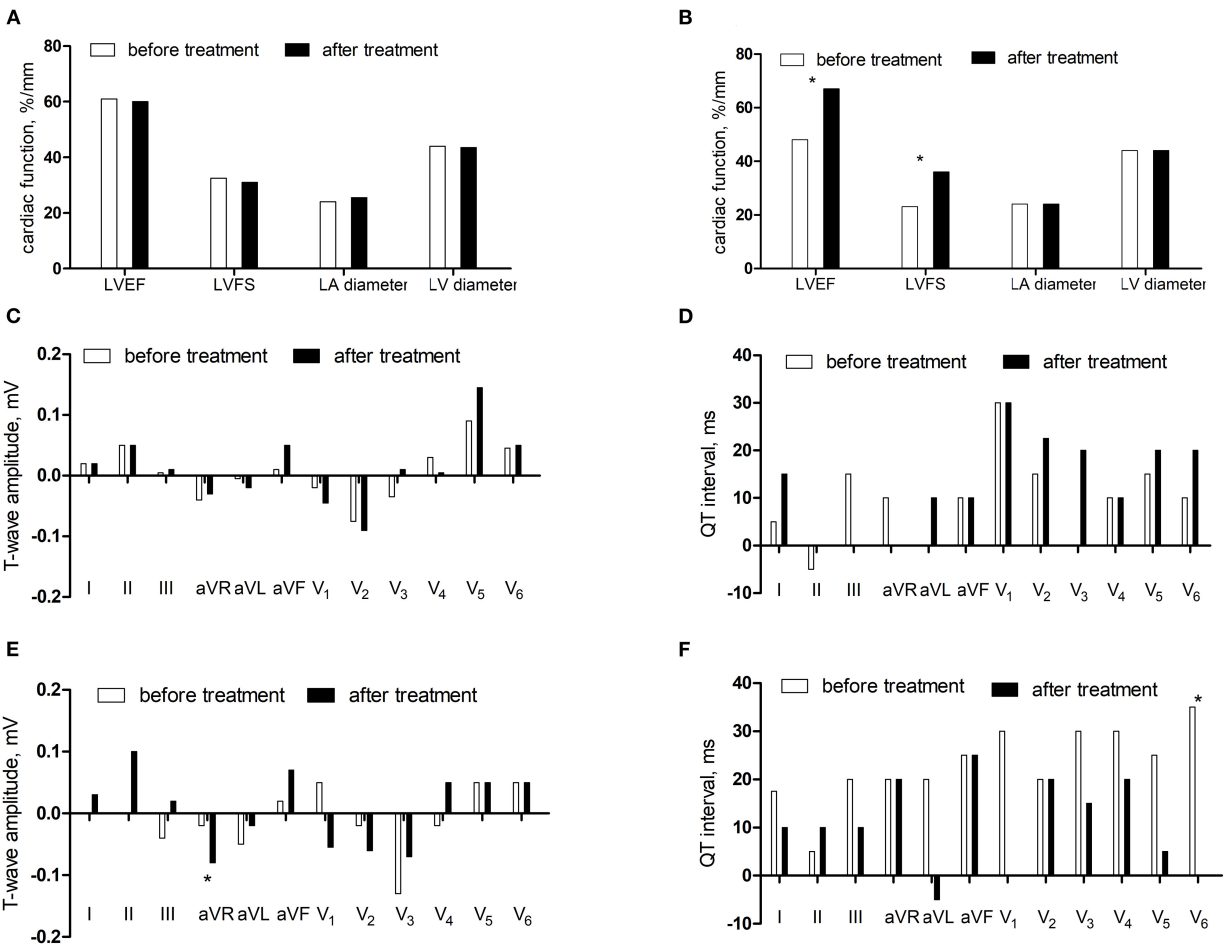
The comparisons before and after treatment. **(A)** The cardiac function in the non-response group. **(B)** The cardiac function in the response group. **(C)** The differences of T-wave amplitude in the non-response group. **(D)** The differences of QT interval in the non-response group. **(E)** The differences of T-wave amplitude in the response group. **(F)** The differences of QT interval in the response group.*P < 0.05.

We followed up 21 children with DCM for 3–25 months, with an average of 10 ± 6 months. After treatment, 9 patients (42.9%) responded, and 12 patients (57.1%) did not respond. There was no statistical difference in LVEF, FS, and inner diameter of left atrium and left ventricle in the non-response group between after treatment and before treatment (*P* > 0.05). LVEF increased and FS expanded in the response group after treatment (*P* < 0.05). There was no statistical difference in the differences of T-wave amplitude and QT interval in the non-response group (*P* > 0.05). The differences of T-wave amplitude in lead aVR increased and the QT interval in lead V_6_ decreased after treatment in the reaction group (*P* < 0.05) (Figure 5).

**Figure 6:**
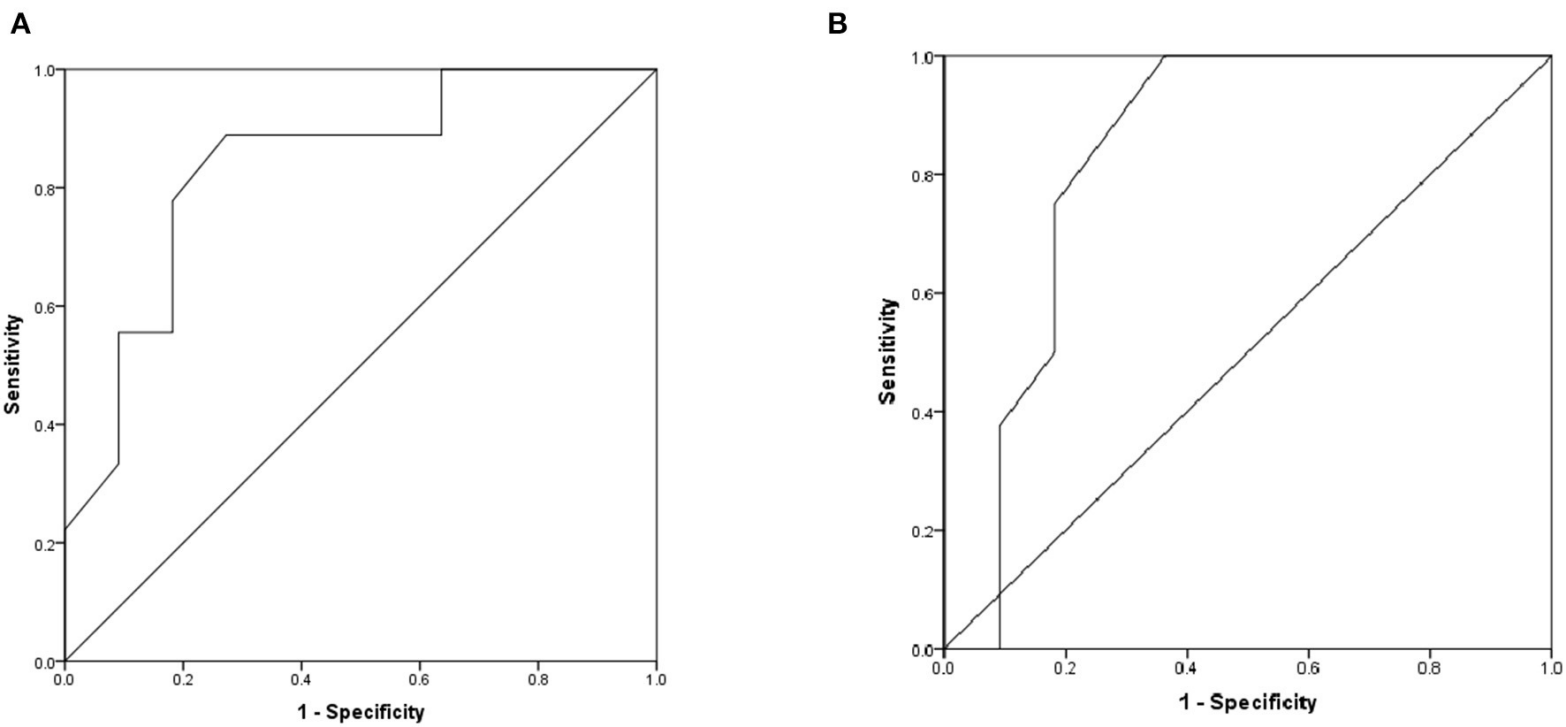
The ROC curve of predictive values on DCM prognosis. **(A)** The difference of T-wave amplitude difference in lead aVR before and after treatment. **(B)** The difference of QT interval difference in lead V_6_ before and after treatment.

For further verification, the comparisons of the differences of T-wave amplitude difference in lead aVR (Z = −2.515, *P* < 0.05) and QT interval difference in lead V_6_ (Z = −2.442, *P* < 0.05) before and after treatment in the non-responding group and the responding group showed statistical significance. The area under the ROC curve of difference of T-wave amplitude difference in lead aVR was 0.838 (*P* < 0.05), the best cutoff value was −0.02 mV, and the sensitivity and specificity were 88.9 and 72.7%, respectively. The area under the ROC curve of the QT interval difference in lead V_6_ was 0.835 (*P* < 0.05), the best cutoff value was 5 ms, and the sensitivity and specificity were 100 and 63.6%, respectively (Figure 6).

For clinical application, we set the value of the differences of T-wave amplitude difference in lead aVR and QT interval difference in lead V_6_ which were −0.05 mV and 5 ms, respectively. The sensitivity and specificity of the combined index were 44.4 and 83.3%, respectively.

## Discussion

DCM has the characteristics of left ventricular dilation and systolic dysfunction, mainly manifested as heart failure. The autonomic nervous system forms interacting feedback loops. When hemodynamic changes occur, the excitability of the sympathetic and parasympathetic nerves changes, then the cardiac output and mean arterial pressure through reflexes to maintain circulatory stability (17, 18).

The action potential of cardiomyocytes and their ionic current are the basis for the formation of cardiac current. Depolarization sequence and repolarization sequence link action potential to surface ECG. The sequence of ventricular depolarization corresponds to the QRS wave in ECG. The sequence of ventricular repolarization corresponds to the ST segment and the T-wave in ECG (19–21). The main depolarizing currents are the inward sodium current and the inward L-type calcium current. The main repolarizing currents are the inward rectifying potassium current (I_k1_) and the rapid (I_Kr_) and slow components (I_Ks_) of the delayed rectifier current. The duration and shape of the action potential are determined by the balance between those depolarizing and repolarizing currents, their time, and their voltage characteristics (22–24).

Our paper shows that in supine ECG, the T-wave amplitudes in leads I, II, and III, aVR, aVF, V_1_, V_4_, V_5_, and V_6_ were lower in the research group than in the control group. In orthostatic ECG, the T-wave amplitude in leads I and II, aVR, aVF, V_1_, V_4_, V_5_, and V_6_ were lower in the research group than in the control group and the QT intervals in lead II, aVR, aVL, V_2_, and V_5_ were longer in the research group than in the control group. This may be related to myocardial fibrosis and myocardial ischemia. Myocardial fibrosis can reduce the conduction of action potentials and interfere with myocardial electrophysiology and cause arrhythmia (25). Fozzard and Makielski (26) and Klabunde (27) reported that when myocardial ischemia occurred, the fast Na^+^ channel was inactivated, the action potential conduction velocity was reduced, and repolarization direction changed, leading to extension of T-wave inversion and QT interval. Ryerson and Giuffre (28) found that 36% of children with DCM had a longer corrected QT interval, and the 50-month survival rate of children with a longer corrected QT interval was about 50%. Chen et al. (29) reported that children with DCM had longer corrected JT interval (49%), corrected QT interval (53%), and abnormal T-wave (low level accounted for 22%, inversion accounted for 10%). In addition, children with abnormal T-wave inversion have a 12-fold higher risk of fatal arrhythmia.

Our study found that compared with supine ECG, the amplitudes of T-wave in lead II, aVR, aVF, V_5_, and V_6_ decreased and the QT intervals in leads I and III, aVF, V_1_, V_2_, V_3_, V_4_, V_5_, and V_6_ shortened in orthostatic ECG in both groups, which may be related to the regulation of cardiovascular activity. When the supine position was turned to the orthostatic position, the blood flow to the heart was reduced and hemodynamic changes occurred. At the same time, the body also can maintain cardiac output and normal cardiovascular activities through neuromodulation, body fluid regulation, and heart self-regulation. Sympathetic nerve activation was the fastest regulatory mechanism when the body blood flow changed (30). The changes in autonomic nerve excitation affect the process of cardiac depolarization and repolarization and result in the changes of ECG waveforms. The T-wave reflects the process of ventricular repolarization, corresponding to the third stage of action potential, and the QT interval reflects the period of cardiac depolarization and repolarization, corresponding to the 0–3 stage of action potential. Excitement, panic, worry, and posture change can all cause sympathetic excitement. Markendorf et al. (31) reported that when the supine position was turned to the orthostatic position, the heart rate and the rate of T-wave inversion increased. Obata et al. (32) reported that the QT interval was lower in orthostatic ECG than supine ECG (366.40 ± 35.00 ms vs. 382.30 ± 33.90 ms, *P* < 0.05).

The distribution of autonomic nerves is unevenly. The sympathetic nerves innervate all parts of the heart. The density around the sinoatrial node and coronary sinus is abundant and gradually decreases from the ventricle bottom to the apex (33, 34). Ng et al. (35) found that the stimulation of sympathetic nerves can significantly shorten the action potential duration, and I_ks_ inhibitors can inhibit this effect. Excitement of the sympathetic nerve can release norepinephrine and then increase the heart rate, conduction velocity, and contractility *via* the pathway of the G-protein, AC (adenylate cyclase), and cAMP (cyclic adenosine monophosphate) to PKA (protein-kinase A), which phosphorylates membrane proteins, including Ca^2+^-handling proteins and ion channels (36). Marx et al. (37) reported that sympathetic excitation activated β-adrenergic receptors to produce PKA, and PKA increased the current amplitude by phosphorylating the hKCNQ1 subunit of the I_ks_ channel through Yotiao protein. When hKCNQ1 gene mutation was destroyed, the I_ks_ channel decreased its AC-mediated response.

Our study also showed that the differences of T-wave amplitudes in leads II and III, aVF, and V_5_ and QT intervals in lead II, aVL, aVF, and V_5_ were lower in the research group than in the control group, which may be related to multiple factors such as myocardial injury and dysfunction of autonomic nervous function. Stimulation of the sympathetic nervous system can cause a cardiac hypertrophy response. When increasing levels of catecholamines stimulate β-adrenergic receptor signaling pathways, the hypertrophic response also causes the activation of many signaling pathways. The continuous activation of the β-adrenergic receptor system combined with the biochemical changes produced by the hypertrophy process activates the desensitization and downregulation pathways and ultimately leads to the weakened β-adrenergic receptor function and loss of contractility (38). In heart failure, the concentration of norepinephrine is decreased (39); cardiac β-adrenergic receptor number, density, and activity are reduced (40); and AC becomes downregulated, which can limit steps in the signaling pathway (41). In addition, G-protein receptor kinases are significantly upregulated and activated in heart failure, which desensitizes the β-adrenergic receptor signal transduction (42, 43). These alterations can be regarded as protecting effects, which save cardiac reserves *via* preventing arrhythmia, apoptosis, and cardiac hypertrophy. Logistic regression analysis showed that the OR value of T-wave amplitude difference in lead III was 0.000, which was a protective factor and the OR value of QT interval difference in lead aVL was almost equal to 1.000, which was an irrelevant factor. The reason for this result was not clear, and further verification was needed. We would expand the sample size and conduct further clinical studies on this. When the differences of T-wave amplitude in lead III and QT interval in lead aVL were 0.00 mV and 30 ms. The sensitivity and specificity of the combined index were 37.5 and 83.6%.

Follow-up was conducted on 21 children with DCM. After treatment, 9 patients (42.9%) responded and 12 patients (57.1%) did not respond. The difference of T-wave amplitude in lead aVR increased and the difference of QT interval in lead V_6_ decreased after treatment in the reaction group, which may be related to the improvement of cardiac ischemia and cardiac function and the enhancement of sympathetic nerve function. ROC showed that differences of T-wave amplitude difference in lead aVR and QT interval difference in lead V_6_ had estimated values for DCM prognosis. When the combined index of differences of T-wave amplitude difference in lead aVR and QT interval difference in lead V_6_ were −0.05 mV and 5 ms, respectively, the sensitivity and specificity of estimating the prognosis of DCM are 44.4 and 83.3%, respectively.

## Conclusions

The differences of T-wave amplitude and QT interval may have a certain value in estimating DCM diagnosis and prognosis.

## Study Limitation

It should be mentioned that the DCM children's sample size of this study is insufficient, the number of children who completed the follow-up is low, and the cause of the differences of T-wave amplitude and QT interval between supine and orthostatic ECG in DCM is not clear. In addition, this study does not analyze the etiology of children with DCM. In the future, we will use gene sequencing and other methods to explore and supplement the etiology of DCM and we will further explore the relationship between postural changes and the release of norepinephrine and whether the sympathetic effect of DCM is related to cardiac function and disease course from the cellular and molecular levels and electrophysiology.

## Significance

This paper is the first to evaluate DCM in children by using the changes of T-wave amplitude and QT interval between the supine and orthostatic ECG. The method is simple and feasible, and it can be used as a marker to evaluate autonomic nervous function in children with DCM.

## Data Availability Statement

The original contributions presented in the study are included in the article/supplementary materials, further inquiries can be directed to the corresponding author/s.

## Ethics Statement

The studies involving human participants were reviewed and approved by Ethical clearance was obtained from the The Second Xiangya Hospital, Central South University. During data collection, written informed consent was obtained from each child family after briefly explaining the purpose, risk, and benefit of the study. All the procedure and purpose were told to the child, and verbal assent was also obtained from each child before any data collection and anthropometric measurements. Confidentiality of data was maintained by avoiding personal identifiers. Written informed consent to participate in this study was provided by the participants' legal guardian/next of kin.

## Author Contributions

CT and CW designed the study, analyzed the data, and drafted and revised the manuscript. YC, SW, and QJ collected the data. FL, YW, and RZ revised the manuscript. CW and XY supervised the execution of the study, checked the data analysis, contributed to the final approval of the manuscript. All the authors contributed to the article and approved the submitted version.

## Conflict of Interest

The authors declare that the research was conducted in the absence of any commercial or financial relationships that could be construed as a potential conflict of interest.

## Abbreviations

DCM: dilated cardiomyopathy
ECG: electrocardiogram
LA: left atrium, LV, left ventricular
LVEF: left ventricular ejection fraction
FS: fractional shortening.
